# Association analyses of apolipoprotein E genotypes and cognitive performance in patients with Parkinson’s disease

**DOI:** 10.1186/s40001-024-01924-2

**Published:** 2024-06-16

**Authors:** Shi-Guo Zhu, Zhu-Ling Chen, Ke Xiao, Zi-Wei Wang, Wen-Bin Lu, Rong-Pei Liu, Shi-Shi Huang, Jian-Hong Zhu, Xiong Zhang, Jian-Yong Wang

**Affiliations:** 1 Department of Neurology, Institute of Geriatric Neurology, the Second Affiliated Hospital and Yuying Children’s Hospital, Wenzhou Medical University, Wenzhou, 325027 Zhejiang China; 2 Department of Preventive Medicine, Institute of Nutrition and Diseases, Wenzhou Medical University, Wenzhou, 325035 Zhejiang China

**Keywords:** Parkinson’s disease, Apolipoprotein E, Genotype, Cognitive dysfunction, Non-motor symptoms

## Abstract

**Background:**

Cognitive impairment is a common non-motor symptom of Parkinson’s disease (PD). The apolipoprotein E (APOE) ε4 genotype increases the risk of Alzheimer’s disease (AD). However, the effect of APOEε4 on cognitive function of PD patients remains unclear. In this study, we aimed to understand whether and how carrying APOEε4 affects cognitive performance in patients with early-stage and advanced PD.

**Methods:**

A total of 119 Chinese early-stage PD patients were recruited. Movement Disorder Society Unified Parkinson’s Disease Rating Scale, Hamilton anxiety scale, Hamilton depression scale, non-motor symptoms scale, Mini-mental State Examination, Montreal Cognitive Assessment, and Fazekas scale were evaluated. APOE genotypes were determined by polymerase chain reactions and direct sequencing. Demographic and clinical information of 521 early-stage and 262 advanced PD patients were obtained from Parkinson’s Progression Marker Initiative (PPMI).

**Results:**

No significant difference in cognitive performance was found between ApoEε4 carriers and non-carriers in early-stage PD patients from our cohort and PPMI. The cerebrospinal fluid (CSF) Amyloid Beta 42 (Aβ42) level was significantly lower in ApoEε4 carrier than non-carriers in early-stage PD patients from PPMI. In advanced PD patients from PPMI, the BJLOT, HVLT retention and SDMT scores seem to be lower in ApoEε4 carriers without reach the statistical significance.

**Conclusions:**

APOEε4 carriage does not affect the cognitive performance of early-stage PD patients. However, it may promote the decline of CSF Aβ42 level and the associated amyloidopathy, which is likely to further contribute to the cognitive dysfunction of PD patients in the advanced stage.

## Background

Parkinson’s disease (PD) is a common neurodegenerative disease with bradykinesia, rigidity, and rest tremor as its key features. Non-motor symptoms develop with PD progression and greatly affect the quality of life [1]. Cognitive impairment is one of the most common non-motor symptom of PD, with a prevalence between 24 to 31% [2], and up to 80% of patients develop dementia during the course of the disease [3, 4].

PD associated cognitive impairment mainly involves executive and visuospatial functions. The mechanisms underlying cognitive decline in PD patients are poorly understood. Multiple brain regions, transmitters, environmental and genetic factors are involved in the development of the cognitive impairment [5]. Variations in genes glucosylceramidase (*GBA*), microtubule-associated protein tau (*MAPT*), leucine-rich repeat serine/threonine-protein kinase 2 (*LRRK2*), α-synuclein (*SNCA*) and apolipoprotein E (*APOE*) are believed to impact cognition in PD patients [6].

APOE is an important protein related to lipid metabolism in mammals. Its gene is polymorphic, with three major alleles ε2, ε3 and ε4 [7]. It is generally accepted that the APOEε4 allele increases the risk of Alzheimer's disease (AD) [8, 9]. Studies also showed that APOEε4 burden is associated with cognitive decline and severity of AD pathologic features in PD patients [10, 11]. However, the conclusion is inconsistent. A study including 447 PD patients did not find a relationship between APOEε4 carriage and cognitive impairment in PD [12].

Some potential reasons for such inconsistent results should be considered. White matter hyperintensity (WMH) is common in the elderly, and it contributes to cognitive impairment in AD and PD [13, 14]. Therefore, WMH is a confounding factor when assessing PD-related cognitive impairment, which is often ignored in previous studies. Furthermore, differences in disease stages among PD patients from different studies may also be the reason for the inconsistent results.

In this study, we aimed to investigate the effect of APOEε4 on cognitive impairment in an early-stage PD cohort after adjusting the influence of WMHs. We also analyzed the relationship between carrying APOEε4 and cognitive decline in early-stage and advanced PD patients from Parkinson’s Progression Marker Initiative (PPMI).

## Methods

### Patients

A total of 119 PD patients of Han Chinese ethnicity were recruited from Department of Neurology, the Second Affiliated Hospital and Yuying Children’s Hospital from July 2020 to May 2023. The patients included 67 men and 52 women, with mean age of 65.04 ± 8.46. All patients were diagnosed according to the movement disorder society (MDS) clinical diagnostic criteria for PD [15]. To better reflect the status of the early stage, only PD patients with Hoehn and Yahr stage ≤ 2, disease duration ≤ 4 years, and without motor complications were included. Because some hereditary PD patients who carry certain mutated genes (such as *SNCA* and *GBA*) are more susceptible to cognitive impairment, patients with family history of PD or secondary and atypical PD were excluded from the study. This study was approved by the Ethics Committee of The Second Affiliated Hospital and Yuying Children’s Hospital, Wenzhou Medical University. All participants signed written informed consents.

### Clinical evaluations

Demographic and clinical information including age, age at onset, gender, disease duration, education history and levodopa equivalent daily doses (LEDD) were collected by face-to-face interview. Neurological and neuropsychological test including Hoehn and Yahr scale, MDS-Unified Parkinson’s Disease Rating Scale (MDS-UPDRS), Hamilton anxiety scale (HAMA), Hamilton depression scale (HAMD), non-motor symptoms scale (NMSS), Mini-mental State Examination (MMSE), and Montreal Cognitive Assessment (MoCA) were assessed by well-trained investigators. All patients were evaluated in the “off” medication state [16]. Fazekas scale [17] was used to quantify the white matter hyperintense lesions in fluid attenuated inversion recovery magnetic resonance imaging (MRI) of the brain.

### Genotyping

Genomic DNA was extracted from peripheral blood samples of PD patients. Polymerase chain reactions (PCRs) were carried out by using a commercial PCR kit (Qingke, Beijing, China) according to the manufacturer’s protocol. DNA fragment containing single nucleotide polymorphisms (SNPs) rs429358 and rs7412 was amplified by using primer pairs 5ʹ-ACT GGA GGA ACA ACT GAC CCC-3ʹ and 5ʹ-CGC TCC TGT AGC GGC TGG-3ʹ. Then, the 2 SNPs were determined by direct sequencing (Qingke, Beijing, China). The ε2, ε3 and ε4 alleles of APOE were identified based on rs429358 and rs7412 as previously described [7]. Each of the patients was classified into subtypes of ApoEε4 carriers and ApoEε4 non-carriers based on the APOE genotypes.

### PPMI cohort

The PPMI is an observational international multicenter study aimed at exploring the relationship between biological markers and PD progression [18]. Demographic information, APOE genotypes, cerebrospinal fluid (CSF) biomarkers, neurological and neuropsychological test including MDS-UPDRS, MoCA, Benton Judgment of Line Orientation Test (BJLOT), Hopkins Verbal Learning Test-revised (HVLT), Letter–Number Sequencing (LNS), Semantic fluency, Symbol digit modalities test (SDMT), Scales for Outcomes in Parkinson’s disease-Autonomic (SCOPA), State–trait anxiety inventory (STAI), and Geriatric Depression Scale (GDS) were obtained from the PPMI database (https://www.ppmi-info.org/access-data-specimens/download-data/). Consistent with our cohort, PD patients meeting Hoehn and Yahr stage ≤ 2, disease duration ≤ 4 years, and without motor complications were defined as early stage.

### Statistical analysis

Statistical analyses were performed using the statistical package of Predictive Analytics Software 19.0. Chi-square test was performed to compare categorical variables between groups. Mann–Whitney *U* test or independent samples *t*-tests was performed to compare continuous variables between groups according to the normality. Linear regression analysis was used to study the effect of APOEε4 on MDS-UPDRS scores after adjusting the age, sex and disease duration. The effect of APOEε4 on cognitive performance was analyzed after adjusting the age, sex, disease duration, education history and Fazekas score. A two-tailed *P* < 0.05 was considered statistically significant.

## Results

### Demographic and clinical data of PD patients

The 119 early-stage PD patients with Hoehn and Yahr stage ≤ 2 comprised 98 ApoEε4 non-carriers and 21 ApoEε4 carriers. Age, age at onset, gender, disease duration, education history, LEDD, Hoehn and Yahr stage and Fazekas scores were comparable between two groups (*P* > 0.05; Table 1). Multivariate analysis after adjusting age, sex and disease duration showed that carrying ApoEε4 was not significantly associated with MDS-UPDRS, NMSS, HAMA and HAMD scores in the early-stage PD patients (*P* > 0.05; Table 1). In consideration of the impact of education and cerebral ischemia on cognition, we further adjusted the years of education and Fazekas score. However, the results also suggested no significant effect of ApoEε4 carriage on MMSE and MOCA scores, as well as their domains (*P* > 0.05; Table 1).

**Table 1:** Demographic and clinical characteristics of PD patients

	ApoEε4 non-carriers	ApoEε4 carrier	*P*	*B*	P^*^
Subject, *n* (%)	98 (82.4)	21 (17.6)	–	–	-
Characteristics					
Age, mean ± SD	65.41 ± 8.52	63.33 ± 8.18	0.310^a^	–	–
Age at onset, mean ± SD	63.37 ± 8.48	61.14 ± 8.06	0.274^a^	–	–
Gender, F/M	44/54	8/13	0.568^b^	–	–
Duration, years (IR)	2.00 (1.00–3.00)	2.00 (1.00–3.00)	0.548^c^	–	–
Education history, years (IR)	5.00 (2.50–8.00)	5.00 (0–8.00)	0.624^c^	–	–
LEDD, mg/day (IR)	0 (0–206.25)	0 (0–212.50)	0.803^c^	–	–
Hoehn and Yahr, I/II	31/67	7/14	0.879^b^	–	–
Fazekas	2.00 (1.00–4.00)	3.00 (1.75–4.00)	0.606^c^	–	–
Motor and non-motor symptoms					
MDS-UPDRS total, mean ± SD	44.39 ± 20.28	47.76 ± 21.87	0.496^a^	0.891	0.439^d^
MDS-UPDRS I, mean ± SD	8.23 ± 4.93	9.24 ± 5.10	0.398^a^	0.231	0.437^d^
MDS-UPDRS II, mean ± SD	9.99 ± 5.64	11.33 ± 6.89	0.344^a^	0.311	0.361^d^
MDS-UPDRS III, mean ± SD	25.57 ± 13.68	26.81 ± 13.50	0.707^a^	0.389	0.607^d^
HAMD, (IR)	6.00 (3.00–10.00)	6.00 (2.00–10.00)	0.986^c^	− 0.107	0.770^d^
HAMA, mean ± SD	8.54 ± 5.72	8.67 ± 5.03	0.923^a^	− 0.060	0.855^d^
NMSS, (IR)	22.50 (12.00–43.00)	35.00 (12.50–46.00)	0.481^c^	0.431	0.806^d^
Cognitive testing					
MMSE	27.00 (23.00–29.00)	28.00 (24.50–29.00)	0.648^c^	0.072	0.807^e^
Orientation, (IR)	10.00 (8.00–10.00)	10.00 (10.00–10.00)	0.086^c^	0.142	0.282^e^
Memory, (IR)	6.00 (5.00–6.00)	5.00 (4.00–6.00)	0.512^c^	− 0.078	0.316^e^
Attention, (IR)	4.00 (3.00–5.00)	5.00 (3.00–5.00)	0.563^c^	0.019	0.849^e^
Language, (IR)	7.00 (6.00–8.00)	7.00 (6.00–8.00)	0.812^c^	0.048	0.618^e^
Construction, (IR)	1.00 (0–1.00)	1.00 (0–1.00)	0.571^c^	0.028	0.361^e^
MoCA, mean ± SD	21.51 ± 5.71	21.93 ± 5.39	0.796^a^	− 0.356	0.340^e^
Memory, mean ± SD	8.32 ± 2.59	8.20 ± 1.97	0.868^a^	− 0.194	0.275^e^
Visuospatial, mean ± SD	2.26 ± 1.32	2.47 ± 1.51	0.612^a^	− 0.051	0.575^e^
Language, (IR)	4.00 (3.50–5.00)	5.00 (3.00–5.00)	0.561^c^	− 0.035	0.708^e^
Attention, (IR)	4.00 (3.00–4.00)	4.00 (3.00–4.00)	0.846^c^	− 0.028	0.659^e^
Executive, (IR)	2.00 (1.00–3.00)	2.00 (2.00–3.00)	0.195^c^	0.026	0.750^e^

*ApoE* apolipoprotein E, *F* female, *HAMA* Hamilton anxiety scale, *HAMD* Hamilton depression scale, *IR* interquartile range, *LEDD* l-dopa equivalent daily dosage, *M* male, *MDS-UPDRS* Movement Disorder Society-Unified Parkinson’s disease Rating Scale, *MMSE* Mini-mental State Examination, *MoCA* Montreal Cognitive Assessment, *PD* Parkinson’s disease, *SD* standard deviation

^*^Analyzed by multiple linear regression

^a^Analyzed by unpaired two-tailed *t*-test

^b^Analyzed by Chi-square test

^c^Analyzed by Mann–Whitney *U* test

^d^Adjusted with age, sex and duration

^e^Adjusted with age, sex, duration, education history and Fazekas score

### Demographic and clinical data in PPMI cohorts

A total of 521 early-stage and 262 advanced PD patients were enrolled in our study. They were assigned to ApoEε4 carriers and ApoEε4 non-carriers groups, respectively.

The early-stage PD patients comprised 398 ApoEε4 non-carriers and 123 ApoEε4 carriers. Education history, Hoehn and Yahr stage distribution, and MDS-UPDRS were comparable between the two groups (*P* > 0.05; Table 2). In contrast, significant differences were present in age (*P* = 0.019), age at onset (*P* = 0.027), gender (*P* = 0.029) and disease duration (*P* = 0.030). Multivariate analysis after adjusting age, sex and disease duration showed that CSF Amyloid Beta 42 (Aβ42) level was significantly lower in ApoEε4 carrier group than in ApoEε4 non-carriers group (655.10 interquartile range, 507.15–899.70 vs. 881.30 interquartile range, 678.00–1207.00; *P* < 0.001; Table 2). While no significant difference was found in α-synuclein level, phosphorylated tau (P-tau) level, total tau (T-tau) level, Aβ42/T-tau ratio, Aβ42/α-synuclein ratio, P-tau/α-synuclein between the two groups (*P* > 0.05; Table 2). The effect of ApoEε4 carriage on cognitive performance including MoCA, BJLOT, HVLT total recall, HVLT delayed recall, HVLT retention, HVLT recognition discrimination index, LNS, semantic fluency and SDMT was analyzed by linear regression analysis after adjusted with age, sex, disease duration and education history. However, the results showed that none of them displayed significant association with ApoEε4 carriage (*P* > 0.05; Table 2).

**Table 2:** Demographic and clinical characteristics of early-stage PD patients in PPMI cohort

	Subject, *n* (ε4−/ε4+)	ApoEε4 non-carriers	ApoEε4 carrier	*P*	*B*	P^*^
Characteristics						
Age, (IR)	521 (398/123)	63.00 (56.00–69.00)	61.00 (54.00–68.00)	0.019^a^	–	–
Age at onset, (IR)	521 (398/123)	62.00 (54.70–67.40)	59.30 (52.10–66.30)	0.027^a^	–	–
Gender, F/M	521 (398/123)	160/238	87/36	0.029^b^	–	–
Duration, years (IR)	521 (398/123)	1.50 (1.90–2.40)	1.20 (0.80–2.00)	0.030^a^	–	–
Education history, years (IR)	509 (388/121)	16.00 (14.00–18.00)	16.00 (13.00–18.00)	0.663^a^	–	–
Hoehn and Yahr, I/II	521 (398/123)	156/242	53/70	0.441^b^	–	–
Motor and non-motor symptoms						
MDS-UPDRS Total, mean ± SD	520 (397/123)	27.28 ± 11.64	27.12 ± 12.16	0.895^c^	− 0.008	0.980^d^
MDS-UPDRS I, (IR)	521 (398/123)	1.00 (0–2.00)	1.00 (0–2.00)	0.796^a^	0.006	0.881^d^
MDS-UPDRS II, (IR)	520 (397/123)	5.00 (2.00–8.00)	5.00 (3.00–8.00)	0.286^a^	0.099	0.368^d^
MDS-UPDRS III, (IR)	521 (398/123)	19.00 (14.00–26.00)	19.00 (13.00–25.00)	0.371^a^	− 0.115	0.622^d^
CSF biomarkers						
α-Synuclein, pg/ml (IR)	404 (306/98)	1429.95 (1078.60–1852.48)	1275.00 (943.30–1708.95)	0.030^a^	− 36.466	0.060^d^
Aβ42, pg/ml (IR)	400 (303/97)	881.30 (678.00–1207.00)	655.10 (507.15–899.70)	< 0.001^a^	− 51.195	< 0.001^d^
P-tau, pg/ml (IR)	419 (323/96)	13.36 (11.05–17.09)	14.58 (10.94–18.39)	0.263^a^	0.259	0.087^d^
T-tau, pg/ml (IR)	442 (337/105)	158.60 (126.75–199.35)	158.69 (121.40–212.25)	0.768^a^	1.517	0.356^d^
Aβ42/T-tau, mean ± SD	401 (351/50)	5.54 ± 1.63	5.07 ± 1.67	0.060^c^	− 0.099	0.116^d^
Aβ42/α-synuclein, (IR)	401 (350/51)	0.63 (0.50–0.73)	0.57 (0.49–0.66)	0.092^a^	− 0.011	0.242^d^
P-tau/α-synuclein, (IR)	403 (353/50)	0.01 (0.01–0.01)	0.01 (0.01–0.01)	0.294^a^	− 6.463 × 10^–5^	0.525^d^
Cognitive testing						
MoCA, (IR)	521 (398/123)	27.00 (25.00–29.00)	28.00 (26.00–29.00)	0.124^a^	0.032	0.643^e^
BJLOT, (IR)	521 (398/123)	12.20 (9.60–14.10)	12.20 (9.60–14.00)	0.729^a^	− 0.089	0.265^e^
HVLT total recall, mean ± SD	521 (398/123)	45.45 ± 10.62	43.48 ± 11.45	0.078^c^	− 0.166	0.533^e^
HVLT delayed recall, (IR)	521 (398/123)	45.00 (37.00–55.00)	42.00 (36.00–52.00)	0.182^a^	− 0.133	0.640^e^
HVLT retention, (IR)	521 (398/123)	48.50 (40.00–56.00)	47.00 (39.00–55.00)	0.136^a^	− 0.313	0.315^e^
HVLT recognition discrimination index, (IR)	521 (398/123)	45.00 (37.00–53.00)	45.00 (36.00–53.00)	0.433^a^	− 0.203	0.493^e^
LNS, (IR)	521 (398/123)	11.50 (10.00–13.00)	11.00 (10.00–13.00)	0.807^a^	0.029	0.692^e^
Semantic fluency, mean ± SD	521 (398/123)	50.38 ± 10.77	51.50 ± 9.48	0.302^c^	0.314	0.261^e^
SDMT, (IR)	521 (398/123)	41.00 (33.00–47.00)	41.00 (33.00–46.00)	0.808^a^	− 0.142	0.540^e^

*Aβ42* amyloid beta 42, *ApoE* apolipoprotein E, *BJLOT* Benton Judgment of Line Orientation Test, *CSF* cerebrospinal fluid, *F* female, *HVLT* Hopkins Verbal Learning Test-revised, *IR* interquartile range, *LNS* Letter–Number Sequencing, *M* male, *MDS-UPDRS* Movement Disorder Society-Unified Parkinson’s disease Rating Scale, *MoCA* Montreal Cognitive Assessment, *P-tau* phosphorylated tau, *PD* Parkinson’s disease, *PPMI* Parkinson’s Progression Markers Initiative, *SD* standard deviation, *SDMT* symbol digit modalities test, *T-tau* total tau

^*^Analyzed by multiple linear regression

^a^Analyzed by Mann–Whitney *U* test

^b^Analyzed by Chi-square test

^c^Analyzed by unpaired two-tailed *t*-test

^d^Adjusted with age, sex and duration

^e^Adjusted with age, sex, duration and education history

The advance PD patients comprised 205 ApoEε4 non-carriers and 57 ApoEε4 carriers. Age, age at onset, education history, Hoehn and Yahr stage distribution, MDS-UPDRS, SCOPA, STAI and GDS were comparable between the two groups (*P* > 0.05; Table 3). Significant differences were present in gender (*P* = 0.025) and disease duration (*P* = 0.025). Similar to patients with early-stage PD, further results showed that cognitive performances were not associated with ApoEε4 carriage after adjusting age, sex, disease duration and education history. However, the BJLOT, HVLT retention, and SDMT seem to be lower in ApoEε4 carriers, though the differences did not reach the statistical significance (*P* = 0.067, *P* = 0.084 & *P* = 0.062, respectively; Table 3).

**Table 3:** Demographic and clinical characteristics of advanced PD patients in PPMI cohort

	Subject, *n* (ε4−/ε4+)	ApoEε4 non-carriers	ApoEε4 carrier	*P*	*B*	P^*^
Characteristics						
Age, mean ± SD	262 (205/57)	69.86 ± 8.68	69.16 ± 8.95	0.590^a^	–	–
Age at onset, mean ± SD	252 (199/53)	61.91 ± 9.32	62.77 ± 9.16	0.546^a^	–	–
Gender, F/M	262 (205/57)	110/95	21/36	0.025^b^	–	–
Duration, years (IR)	252 (199/53)	7.00 (5.00–11.00)	6.00 (4.00–9.00)	0.025^c^	–	–
Education history, years (IR)	258 (202/56)	16.00 (12.75–18.00)	15.50 (13.25–18.00)	0.799^c^	–	–
Hoehn and Yahr, III/IV/V	262 (205/57)	186/16/3	57/0/0	0.058^b^	–	–
Motor and non-motor symptoms						
MDS-UPDRS Total, mean ± SD	214 (163/51)	56.40 ± 21.12	57.26 ± 16.78	0.792^a^	0.176	0.831^d^
MDS-UPDRS I, (IR)	259 (202/57)	2.50 (1.00–5.00)	3.00 (1.00–5.50)	0.842^c^	0.011	0.931^d^
MDS-UPDRS II, mean ± SD	259 (202/57)	14.92 ± 7.84	15.28 ± 6.78	0.750^a^	0.101	0.737^d^
MDS-UPDRS III, mean ± SD	216 (165/51)	35.72 ± 13.49	36.63 ± 11.33	0.662^a^	0.054	0.920^d^
MDS-UPDRS IV, (IR)	214 (162/52)	3.00 (0–6.00)	4.00 (0–6.00)	0.270^c^	0.205	0.202^d^
Motor fluctuations, mean ± SD	214(162/52)	0.83 ± 1.32	0.87 ± 1.33	0.880^a^	0.034	0.537^d^
Dyskinesias, mean ± SD	214 (162/52)	2.72 ± 3.11	3.27 ± 3.33	0.280^a^	0.171	0.203^d^
SCOPA, (IR)	151 (111/40)	15.00 (9.00–21.00)	13.00 (8.25–19.00)	0.378^c^	− 0.454	0.274^d^
STAI, (IR)	152 (112/40)	91.50 (85.00–97.00)	91.00 (83.00–96.75)	0.400^c^	− 0.386	0.451^d^
GDS, (IR)	152 (112/40)	6.00 (5.00–7.00)	7.00 (4.25–7.75)	0.644^c^	0.008	0.940^d^
Cognitive testing						
MoCA, (IR)	110 (88/22)	26.00 (23.00–28.00)	25.00 (22.75–27.25)	0.556^c^	0.093	0.722^e^
BJLOT, mean ± SD	103 (80/23)	10.12 ± 3.28	8.97 ± 3.81	0.156^a^	− 0.392	0.067^e^
HVLT total recall, mean ± SD	105 (81/24)	45.56 ± 11.11	40.79 ± 13.41	0.082^a^	− 0.626	0.338^e^
HVLT delayed recall, mean ± SD	105 (81/24)	44.91 ± 11.98	39.29 ± 13.57	0.053^a^	− 1.072	0.147^e^
HVLT retention, mean ± SD	105 (81/24)	46.28 ± 12.54	41.08 ± 13.99	0.085^a^	− 1.358	0.084^e^
HVLT recognition discrimination index, mean ± SD	105 (81/24)	46.99 ± 11.74	44.83 ± 13.12	0.444^a^	0.094	0.890^e^
LNS, (IR)	104 (81/23)	10.00 (8.00–13.00)	10.00 (5.00–14.00)	0.376^c^	− 0.220	0.318^e^
Semantic fluency, mean ± SD	105 (81/24)	49.46 ± 12.11	47.75 ± 13.06	0.553^a^	− 0.337	0.662^e^
SDMT, mean ± SD	119 (92/27)	33.52 ± 13.57	26.82 ± 12.56	0.024^a^	− 1.139	0.062^e^

*ApoE* apolipoprotein E, *BJLOT* Benton Judgment of Line Orientation Test, *F* female, *GDS* Geriatric Depression Scale, *HVLT* Hopkins Verbal Learning Test-revised, *IR* interquartile range, *LNS* Letter–Number Sequencing, *M* male, *MDS-UPDRS* Movement Disorder Society-Unified Parkinson’s disease Rating Scale, *MoCA* Montreal Cognitive Assessment, *PD* Parkinson’s disease, *PPMI* Parkinson’s Progression Markers Initiative, *SCOPA* Scales for Outcomes in Parkinson’s disease-Autonomic, *SD* standard deviation, *SDMT* symbol digit modalities test, *STAI* state–trait anxiety inventory

^*^Analyzed by multiple linear regression

^a^Analyzed by unpaired two-tailed *t*-test

^b^Analyzed by Chi-square test

^c^Analyzed by Mann–Whitney *U* test

^d^Adjusted with age, sex and duration

^e^Adjusted with age, sex, duration and education history

## Discussion

Cognitive impairment in PD is common, and multiple environmental and genetic factors are believed to be involved in its pathogenesis. ApoEε4 is an important risk factor for the development of AD, and its role in cognitive dysfunction in PD cannot be ignored. In the current study, we compared the cognitive function of ApoEε4 carriers and non-carriers in a cohort of 119 early-stage PD patients. The results showed no significant difference in cognitive performance between the two groups. We further analyzed the relationship between cognitive function and ApoEε4 carriage in patients with early-stage and advanced PD from PPMI. Consistent with our results, early-stage PD patients who carry ApoEε4 do not experience more significant cognitive decline than non-carriers. However, their CSF Aβ42 level was significantly lower than that of non-carriers. In advanced PD patients, the BJLOT, HVLT retention and SDMT scores seem to be lower in ApoEε4 carriers without reach the statistical significance.

A proportion of PD patients are complicated by cognitive dysfunction, ranging from subjective cognitive decline (SCD) and mild cognitive impairment (MCI) to PD dementia (PDD) [5, 6]. The cognitive changes are global and particularly prominent in executive function, visuospatial ability and Verbal Fluency [5, 19, 20]. The mechanisms underlying PD-related cognitive impairment remain unclear. The most important neuropathology is the deposition of α-synuclein in Lewy bodies, which can spread in a prion-like way, and is accompanied by a series of neurotransmitter changes. Cognitive impairment often occurs when the pathology affects the neocortical and limbic lobes [5, 6]. Interestingly, studies showed that coexisting AD pathology contribute to the development of cognitive decline in PD [21, 22].

ApoEε4 is the strongest genetic risk factor for late-onset AD. Studies showed that APOEε4 is associated with cerebral Aβ deposition in AD and non-AD dementia patients [23]. It also contributes to the development of cognitive dysfunction by affecting tau-induced neurodegeneration, glia reactions, and blood–brain barrier [9]. Furthermore, recent studies found that APOEε4 can exacerbate α-synuclein pathology and accelerate cognitive decline [24–26]. Therefore, it is of great value to explore the relationship between APOE status and cognitive function in PD patients.

So far, many studies have explored the relationship between APOE polymorphisms and PD-related cognitive impairment. But the results are controversial. Some studies showed that APOEε4 carriage increases the risk of cognitive impairment in PD patients [10, 27], while other studies denied the association [12, 28]. Heterogeneity among PD patients is a factor that must be taken into consideration. On the other hand, WMHs are a common finding on brain MRI of elderly, which are often thought to aggravate cognitive impairment in patients with PD and AD [13, 14]. It is worth noting that previous studies exploring the relationship between APOEε4 and cognitive impairment in PD did not consider WMHs as a confounding factor. Therefore, in the current study, we included only early-stage PD patients and adjusted WMHs by using Fazekas scale in the analysis. The results showed that in early-stage PD patients, carrying APOEε4 did not affect cognitive performance. Similar results were found in early-stage PD patients from PPMI, although they did not correct the Fazekas score due to the lack of data. It should be taken into consideration that the cognitive battery of tests used for the Chinese cohort (MoCA and MMSE) were not the same as for the PPMI cohort, which included the MoCA, BJLOT, HVLT, LNS, Semantic fluency, and SDMT. This difference could induce a bias in underestimating minor cognitive impairment in the early Chinese cohort. Additionally, the MoCA scores of early PD patients in the Chinese population were lower than those from PPMI cohort, which may be result from the shorter education duration in our population.

Highlighted should be that we found the CSF Aβ42 level is lower in PD patients with APOEε4 even in the early stage, though no cognitive changes were observed at that time. Lower CSF Aβ42 level is linked to cortical Aβ plaques and is a core biomarker for AD and MCI due to AD [29]. Researches showed that decreased CSF Aβ42 can be detected in the preclinical stage of AD patients [30]. Previous studies also showed that PD patients who carry APOEε4 have lower CSF Aβ42 level and worse cognitive function than non-carriers [31, 32]. Our study further demonstrates the impact of APOEε4 on CSF Aβ42 in PD patients even in the absence of obvious cognitive impairment. It would be interesting to further explore relationship between APOE genotypes, CSF Aβ42, and cognitive changes in patients with advanced PD. Unfortunately, only 24 patients with advanced PD from PPMI had CSF biomarkers measured.

Advanced stage of PD usually means patients have more complex symptoms and greater impairment in their daily life. This stage is often defined in practice according to the Hoehn and Yahr stage, duration of the disease, motor complications, and non-motor symptoms [33–36]. In this study, we defined PD patients who met Hoehn and Yahr stage ≤ 2, disease duration ≤ 4 years, and without motor complications as early stage, and those who did not meet these criteria as advanced stage. To further clarify whether the effect of APOEε4 on CSF Aβ42 further affects the cognition function of PD patients, we analyzed the relationship between APOEε4 carrying and cognitive performance in advanced PD patients from PPMI. Our results indicated that APOEε4 carriers seem to have lower BJLOT, HVLT retention and SDMT scores, although the differences did not reach the traditional statistical significance, which is probably due to the relatively small sample size. The lack of PD patients with Hoehn and Yahr stage IV and V in APOEε4 group and difference in disease duration between the two groups were a pity. Our results suggest that visuospatial judgment, memory and information processing speed reflected by BJLOT, HVLT retention and SDMT may be the preferentially affected cognitive domains in PD patients with APOEε4. A real cohort of advanced PD or a longitudinal observation is required to verify the conclusion.

Meanwhile, we have to acknowledge that our study lacks longitudinal observation, the presence of which would otherwise enhance the reliability. Future study with larger population and longitudinal observation are warranted to confirm our findings.

## Conclusions

In conclusion, our study demonstrated that carrying APOEε4 does not affect the cognitive function of early-stage PD patients, but it is associated with a decrease in their CSF Aβ42, which is likely to promote the decline of cognitive function in PD patients in the advanced stage.

## Data Availability

The original contributions presented in the study are included in the article, further inquiries can be directed to the corresponding authors.

## Abbreviations

Aβ42: Amyloid beta 42
AD: Alzheimer’s disease
APOE: Apolipoprotein E
BJLOT: Benton Judgment of Line Orientation Test
CSF: Cerebrospinal fluid
GBA: Glucosylceramidase
GDS: Geriatric Depression Scale
HAMA: Hamilton anxiety scale
HAMD: Hamilton depression scale
HVLT: Hopkins Verbal Learning Test-revised
LEDD: Levodopa equivalent daily doses
LNS: Letter–Number Sequencing
LRRK2: Leucine-rich repeat serine/threonine-protein kinase 2
MAPT: Microtubule-associated protein tau
MCI: Mild cognitive impairment
MDS: Movement Disorder Society
MDS-UPDRS: MDS-Unified Parkinson’s Disease Rating Scale
MMSE: Mini-mental State Examination
MoCA: Montreal Cognitive Assessment
MRI: Magnetic resonance imaging
NMSS: Non-motor symptoms scale
PCRs: Polymerase chain reactions
PD: Parkinson’s disease
PDD: PD dementia
PPMI: Parkinson’s Progression Marker Initiative
SCD: Subjective cognitive decline
SCOPA: Scales for Outcomes in Parkinson’s disease-Autonomic
SDMT: Semantic fluency, and Symbol digit modalities test
SNCA: α-Synuclein
SNPs: Single nucleotide polymorphisms
STAI: State–trait anxiety inventory
WMH: White matter hyperintensity
